# Cytokine profiles of aqueous humor from sequential bilateral cataract surgery: Impact of diabetes on inflammatory responses

**DOI:** 10.1371/journal.pone.0361977

**Published:** 2026-11-02

**Authors:** Yongseok Mun, Young Joo Shin, Kayoung Yi, Dong Gyu Choi, Dae Joong Ma

**Affiliations:** 1 Department of Ophthalmology, Hallym University Kangnam Sacred Heart Hospital, Seoul, Republic of Korea; 2 Hallym BioEyeTech Research Center, Hallym University, Chuncheon, Republic of Korea; 3 Department of Ophthalmology, College of Medicine, Hallym University, Chuncheon, Republic of Korea; Sanmenxia Central Hospital, Henan University of Science and Technilogy, CHINA

## Abstract

We investigated aqueous humor (AH) cytokine profiles in first- and second-operated eyes of patients undergoing sequential bilateral cataract surgery and explored these profiles according to diabetes mellitus (DM) status. This retrospective, within-subject, comparative observational study included 71 patients, comprising 39 without DM and 32 with DM but without diabetic retinopathy, who underwent uneventful sequential bilateral cataract surgery within a 2-week interval. The concentrations of vascular endothelial growth factor, angiopoietin-2, placental growth factor, interleukin (IL)-6, IL-8, monocyte chemoattractant protein-1 (MCP-1), and transforming growth factor–β2 (TGF-β2) in the AH were analyzed. In patients without DM, mean IL-6 concentrations were nominally lower in the second eye than in the first eye (P = 0.041), although this finding did not remain statistically significant after FDR adjustment. In patients with DM, there were no significant differences in cytokine concentrations between the first and second eyes. The eye-order × DM interaction for IL-6 was not statistically significant (P = 0.644). Compared with patients without DM, higher MCP-1 and TGF-β2 concentrations were observed in the first eye and higher TGF-β2 concentrations in the second eye, with the strongest statistical evidence observed for first-eye TGF-β2, which remained significant after FDR adjustment. Differences in AH cytokine profiles were observed between patients with and without DM during sequential bilateral cataract surgery. The lower mean IL-6 concentration observed in the second eyes of patients without diabetes represents a novel, although exploratory, observation. Whether this finding reflects pre-existing inter-eye variability or a biological response associated with sequential surgery remains to be determined.

## Introduction

Cataract surgery, which contributes significantly to improving the quality of life of patients of all ages, is among the most commonly performed procedures globally [1,2]. Most cataracts are age-related [3], and there are no significant differences in the incidence and progression of cataracts between both eyes. Consequently, second-eye surgeries account for approximately 45% of all cataract procedures [4].

Diabetes mellitus (DM) is also a well-established risk factor for cataract development [5], with studies indicating a 2–5-fold increased incidence in individuals with DM relative to that in the general population [6]. Recent studies have shown that the concentrations of interleukin (IL)-1β, IL-6, IL-8, monocyte chemoattractant protein (MCP)-1, vascular endothelial growth factor (VEGF), and transforming growth factor (TGF)-β1 and TGF-β2 in the aqueous humor (AH) are significantly higher in cataract patients with diabetes mellitus (DM) than in those without DM, even in the absence of diabetic retinopathy (DR) [7–9]. Conversely, the concentrations of IL-2, IL-10, IL-12, TNF-α, and IFN-α were significantly lower in AH of cataract patients with DM compared to those without DM.

Patients tend to experience greater levels of pain during their second cataract surgery performed under topical anesthesia relative to their first surgery [10]. It has been hypothesized that this effect is due to decreased levels of preoperative anxiety [10]. However, studies have also shown that concentrations of MCP-1, TNF-α, and IL-1β are elevated in the AH during second-eye cataract surgery [11–13]. These findings have been proposed to reflect microenvironmental changes in the fellow eye associated with first-eye surgery. Given that these cytokines are known pain-related inflammatory mediators, their increased concentration may also help explain the heightened pain reported during second-eye procedures.

Because both DM and sequential bilateral cataract surgery have been associated with differences in AH cytokine profiles, these profiles may vary according to surgical order and DM status. To further explore this, we investigated AH cytokine profiles in patients with senile cataracts undergoing sequential bilateral cataract surgery, focusing on comparisons according to surgical order and DM status. Given the reported associations between AH cytokine profiles, intraoperative pain, and postoperative complications, this study aimed to investigate differences in AH cytokine profiles between first- and second-operated eyes and to explore these profiles according to diabetes status.

## Materials and methods

### Ethical approval

This retrospective within-subject comparative observational study was conducted at Hallym University Kangnam Sacred Heart Hospital (approval number: 2022-12-020) in accordance with the principles of the Declaration of Helsinki. Written informed consent for AH sample collection and biobanking, including potential use for future research, was obtained from all participants at the time of surgery. The requirement for additional informed consent for the present analysis was waived due to the retrospective nature of the study.

### Patients

This retrospective observational study was conducted at Hallym University Kangnam Sacred Heart Hospital in Seoul, Republic of Korea, between June 2023 and June 2025. The study included all consecutive patients who underwent uneventful sequential bilateral cataract surgery within 2 weeks. Patients with DR or any other ocular surgeries or diseases, except for senile cataracts, were excluded.

The data were accessed for research purposes on October 1, 2025, and the authors did not have access to information that could identify individual participants during or after data collection, as all data and samples were handled in a coded and de-identified manner.

All patients received a comprehensive ophthalmologic evaluation, which included a slit-lamp examination, measurement of intraocular pressure, and a fundus examination. The cataracts were graded by visually assessing the crystalline lens with a slit lamp, and the findings were compared to the Lens Opacities Classification System III (LOCS III) to determine both the type and severity of the cataract [14]. Mixed cataracts were defined as lenses exhibiting more than one cataract subtype with comparable severity based on LOCS III grading.

The decision regarding which eye to operate on first was made at the surgeon’s discretion. In routine clinical practice, the eye with poorer visual acuity was generally selected for the first surgery after discussion with the patient, although other clinical considerations and patient preference were also taken into account when appropriate. Topical 1.5% levofloxacin (CRAVIT; Santen Pharmaceutical, Osaka, Japan) and topical 0.1% bromfenac sodium hydrate (Bronuck; Taejoon Pharmaceutical, South Korea) ophthalmic solutions were instilled in the eye scheduled for surgery starting 3 d before the procedure.

### Sample collection and surgical procedure

AH samples were obtained at the start of cataract surgery by anterior chamber paracentesis using a 30-gauge needle and stored at −80 °C until analysis. All surgeries were performed using standard phacoemulsification techniques with intraocular lens implantation.

### Analysis of cytokines in AH samples

Cytokine analyses were performed retrospectively. The cytokine concentrations in the AH samples were measured using the Luminex system (Luminex xMAP Technology, Bio-Rad Laboratories, Inc., Hercules, CA) according to the manufacturers’ protocols. The following kits were used: the Human XL Cytokine Premixed Kit (FCSTM18, R&D Systems, Minneapolis, MN) for monocyte chemoattractant protein-1 (MCP-1), interleukin (IL)-6, interleukin-8 (IL-8), and vascular endothelial growth factor (VEGF); the Human Premixed Multi-Analyte Kit (LXSAHM, R&D Systems) for placental growth factor (PlGF) and angiopoietin-2; and the TGF-β Premixed Kit (FCSTM17, R&D Systems) for transforming growth factor beta-2 (TGF-β2).

### Statistical methods

Data are expressed as mean ± standard deviation, with cytokine concentrations additionally presented as median (interquartile range), or as frequency and percentage. Snellen corrected distance visual acuity (CDVA) was converted to logMAR values. Comparisons of the first and second eyes were performed using a paired t-test or marginal homogeneity test, depending on the distribution and nature of the variables. Similarly, comparisons of patients with and without DM were conducted using an independent t-test or chi-squared test. Normality of the paired differences between the first- and second-eye cytokine concentrations was assessed using the Shapiro–Wilk test. Wilcoxon signed-rank tests were additionally performed as non-parametric sensitivity analyses of the paired cytokine comparisons. To further evaluate the robustness of the primary findings to deviations from normality, exact paired permutation tests of the mean difference were performed as appropriate. To formally assess whether the first- versus second-eye difference in IL-6 differed according to DM status, a repeated-measures general linear model was performed with eye order (first- versus second-operated eye) as the within-subject factor, DM status as the between-subject factor, and the eye-order × DM interaction term. As an exploratory analysis, the association between the interval between surgeries and the first- versus second-eye difference in IL-6 concentration among patients without DM was assessed using Spearman’s rank correlation analysis. Box plots were used to demonstrate medians, interquartile ranges, and Tukey whiskers.

To account for multiple comparisons across the seven cytokines, P values were additionally adjusted using the Benjamini–Hochberg false discovery rate (FDR) procedure, with the seven cytokines within each corresponding comparison considered as a family of tests. Both nominal and FDR-adjusted P values were considered in the interpretation of the results.

A two-sided P value < 0.05 was considered statistically significant. For the FDR analyses, an adjusted P value < 0.05 was considered statistically significant. All analyses were performed using IBM SPSS Statistics for Windows (version 22.0; IBM Corp., Armonk, NY).

## Results

This study included 142 eyes from 71 patients, comprising 39 patients without DM and 32 patients with DM. Clinical characteristics of patients with DM are summarized in S1 Table. The eyes from each patient were categorized into two groups based on the order of surgery: first eye and second eye.

Baseline demographic and ocular characteristics were comparable between patients with and without diabetes, and no significant differences were observed between first and second eyes across all groups (Table 1). Table 2 shows a comparison of the cytokine concentrations in the AH of the first eyes of the patients with and without DM. The concentrations of MCP-1 and TGF-β2 in the AH of the first eye were nominally higher in patients with DM than in those without DM (P = 0.028 and P = 0.002, respectively). After FDR adjustment, the difference in TGF-β2 remained statistically significant (adjusted P = 0.014), whereas the difference in MCP-1 did not (adjusted P = 0.098). The concentrations of the other cytokines did not significantly differ between patients with and without DM.

**Table 1:** Demographic data for all patients.

	Total (n = 71)		Patients without DM (n = 39)		Patients with DM (n = 32)		*P*-value	
	First-eye	Second-eye	First-eye	Second-eye	First-eye	Second-eye	First-eye	Second-eye
**Age (years)**	71.58 ± 9.16		73.23 ± 8.00		69.56 ± 10.17		0.093^a^	
**Male, n (%)**	34 (47.89%)		20 (51.28%)		14 (43.75%)		0.347^a^	
**Surgical interval (days)**	6.42 ± 4.06		6.23 ± 3.76		6.66 ± 4.46		0.664^a^	
**CDVA (logMAR)**	0.31 ± 0.25	0.27 ± 0.18	0.31 ± 0.29	0.28 ± 0.21	0.32 ± 0.20	0.27 ± 0.13	0.995^a^	0.823^a^
**Axial length (mm)**	23.57 ± 0.97	23.56 ± 1.06	23.51 ± 0.80	23.53 ± 0.91	23.64 ± 1.16	23.61 ± 1.23	0.585^a^	0.736^a^
**ECD (cell/mm**^**2**^)	2634.13 ± 368.37	2583.07 ± 450.25	2634.38 ± 302.64	2617.00 ± 359.42	2633.81 ± 440.62	2541.72 ± 544.25	0.995^a^	0.487^a^
**Type of cataract, n (%)**								
**Nuclear**	12 (16.90%)	14 (19.72%)	8 (20.51%)	8 (20.51%)	4 (12.50%)	6 (18.75%)	0.131^b^	0.155^b^
**Cortical**	2 (2.82%)	2 (2.82%)	1 (2.56%)	1 (2.56%)	1 (3.13%)	1 (3.13%)		
**Posterior subcapsular**	4 (5.63%)	4 (5.63%)	0 (0.00%)	0 (0.00%)	4 (12.50%)	4 (12.50%)		
**Mixed**	53 (74.65%)	51 (71.83%)	30 (76.92%)	30 (76.92%)	23 (71.88%)	21 (65.63%)		

CDVA, corrected distance visual acuity; DM, diabetes mellitus; ECD, corneal endothelial cell density. ^a^Independent t-test between patients without and with DM. ^b^Chi-square test between patients without and with DM.

**Table 2:** Cytokine concentrations in the aqueous humor samples from the first eyes of the patients with and without diabetes mellitus.

Cytokine/chemokine	Patients without DM (n = 39)	Patients with DM (n = 32)	Mean difference (95% CI)	*P*-value
**VEGF (pg/mL)**	117.57 ± 64.71 109.32 (74.25–148.67)	137.02 ± 76.81 104.47 (85.05–166.18)	−19.44 (−53.98–15.08)	0.264
**Angiopoietin-2** **(pg/mL)**	64.65 ± 83.81 29.62 (15.86–50.79)	41.77 ± 1.94 21.61 (10.14–45.70)	19.22 (−39.81–78.25)	0.506
**PlGF (pg/mL)**	2.18 ± 1.91 1.71 (1.15–2.21)	1.90 ± 1.35 1.95 (1.30–2.33)	0.28 (−0.63–1.19)	0.535
**IL-6 (pg/mL)**	19.90 ± 19.89 13.36 (8.33–20.49)	21.12 ± 43.06 11.49 (8.92–17.53)	−1.23 (−17.87–15.42)	0.883
**IL-8 (pg/mL)**	4.24 ± 2.00 4.10 (2.76–5.42)	5.15 ± 2.97 4.33 (3.11–5.65)	−0.90 (−2.13–0.32)	0.146
**MCP-1 (pg/mL)**	320.88 ± 112.41 313.13 (237.88–403.28)	391.71 ± 149.03 323.51 (260.32–391.26)	−70.83 (−133.62 – −8.04)	0.028
**TGF-β2 (pg/mL)**	511.93 ± 502.48 446.15 (136.95–728.85)	1859.80 ± 1998.02 354.59 (171.25–741.21)	−1347.88 (−2155.29 – −540.47)	0.002

Data are presented as mean ± standard deviation and median (interquartile range), as appropriate. IL, interleukin; MCP-1, monocyte chemoattractant protein 1; TGF-β2, transforming growth factor beta 2; VEGF, vascular endothelial growth factor.

Table 3 shows a comparison of the cytokine concentrations in the AH of the second eyes of the patients with and without DM. The concentration of TGF-β2 in the AH of the second eye was higher in patients with DM than in those without DM in the nominal analysis (P = 0.020), although this difference did not remain statistically significant after FDR adjustment (adjusted P = 0.140). However, the concentrations of other cytokines in the AH did not significantly differ between the patients with and without DM.

**Table 3:** Cytokine concentrations in the aqueous humor samples of the second eyes of the patients with and without diabetes mellitus.

Cytokine/chemokine	Patients without DM (n = 39)	Patients with DM (n = 32)	Mean difference (95% CI)	*P*-value
**VEGF (pg/mL)**	118.80 ± 48.81 112.96 (64.84–189.68)	122.69 ± 74.47 94.43 (70.11–176.32)	−3.89 (−35.01–27.23)	0.803
**Angiopoietin-2** **(pg/mL)**	60.70 ± 54.63 40.09 (10.14–43.45)	52.41 ± 37.63 24.10 (10.86–66.57)	4.77 (−45.11–54.65)	0.843
**PlGF (pg/mL)**	1.94 ± 0.75 1.71 (0.90–2.30)	2.38 ± 1.84 1.89 (1.03–2.87)	−0.44 (−1.29–0.41)	0.299
**IL-6 (pg/mL)**	13.54 ± 8.17 12.34 (6.69–19.74)	12.03 ± 9.27 8.95 (4.73–18.46)	1.51 (−3.22–6.23)	0.525
**IL-8 (pg/mL)**	4.61 ± 2.52 4.68 (3.01–5.85)	5.90 ± 6.19 4.10 (2.73–5.96)	−1.29 (−3.52–0.94)	0.253
**MCP-1 (pg/mL)**	336.36 ± 89.33 404.37 (273.63–495.81)	356.95 ± 169.46 390.26 (234.32–479.47)	−20.59 (−87.46–46.28)	0.538
**TGF-β2 (pg/mL)**	710.51 ± 1200.19 1162.16 (251.75–2782.34)	1923.97 ± 2290.49 1187.15 (176.62–3706.03)	−1213.47 (−2221.04 – −205.89)	0.020

Data are presented as mean ± standard deviation and median (interquartile range), as appropriate. IL, interleukin; MCP-1, monocyte chemoattractant protein 1; TGF-β2, transforming growth factor beta 2; VEGF, vascular endothelial growth factor.

Table 4 presents the cytokine concentrations in the AH of the first and second eyes across all patient groups. Overall, the cytokine concentrations did not differ significantly between the first and second eyes. Among patients without DM, mean IL-6 concentrations in the AH were lower in the second eye than in the first eye in the paired t-test analysis (nominal P = 0.041). Although the paired differences in IL-6 concentrations deviated significantly from normality (Shapiro–Wilk test, P < 0.001) and the Wilcoxon signed-rank sensitivity analysis did not reach statistical significance (P = 0.176), the difference in mean IL-6 concentration remained significant in the exact paired permutation test (P = 0.032) (Fig 1A). However, this difference did not remain statistically significant after FDR adjustment (adjusted P = 0.287) and was therefore considered exploratory. A formal repeated-measures analysis showed no significant eye-order × DM interaction for IL-6 (F[1,52] = 0.216, P = 0.644; partial η^2^ = 0.004).

**Table 4:** Cytokine concentrations in the aqueous humor samples from the first and second eyes of all patients.

Cytokine/chemokine	First-eye (n = 71)	Second-eye (n = 71)	Mean difference (95% CI)	*P*-value
**VEGF (pg/mL)**	126.19 ± 70.47 110.50 (68.95–183.88)	120.52 ± 61.05 97.31 (75.82 - 174.12)	5.67 (−6.13–17.46)	0.341
**Angiopoietin 2** **(pg/mL)**	41.79 ± 61.86 31.18 (15.86–43.46)	39.37 ± 44.81 22.86 (11.22 - 45.27)	11.63 (−9.19–32.45)	0.251
**PlGF (pg/mL)**	2.06 ± 1.69 1.71 (1.08–2.21)	2.12 ± 1.31 1.95 (1.28 - 2.46)	0.05 (−0.56–0.65)	0.872
**IL-6 (pg/mL)**	20.36 ± 30.44 13.36 (8.23–20.10)	12.98 ± 8.54 11.35 (7.53 - 17.43)	8.05 (−0.51–16.61)	0.065
**IL-8 (pg/mL)**	4.65 ± 2.51 4.20 (2.92–5.56)	5.14 ± 2.51 4.22 (2.89 - 5.70)	−0.60 (−1.68–0.48)	0.270
**MCP-1 (pg/mL)**	351.68 ± 133.36 336.29 (258.62–450.45)	345.64 ± 130.98 361.65 (251.07 - 440.37)	0.30 (−25.52–26.12)	0.982
**TGF-β2 (pg/mL)**	1128.75 ± 1543.77 537.27 (188.27–1207.55)	1254.48 ± 1858.77 378.99 (180.34 - 1403.41)	−107.39 (−397.03–182.26)	0.461

Data are presented as mean ± standard deviation and median (interquartile range), as appropriate. IL, interleukin; MCP-1, monocyte chemoattractant protein 1; TGF-β2, transforming growth factor beta 2; VEGF, vascular endothelial growth factor.

**Fig 1:**
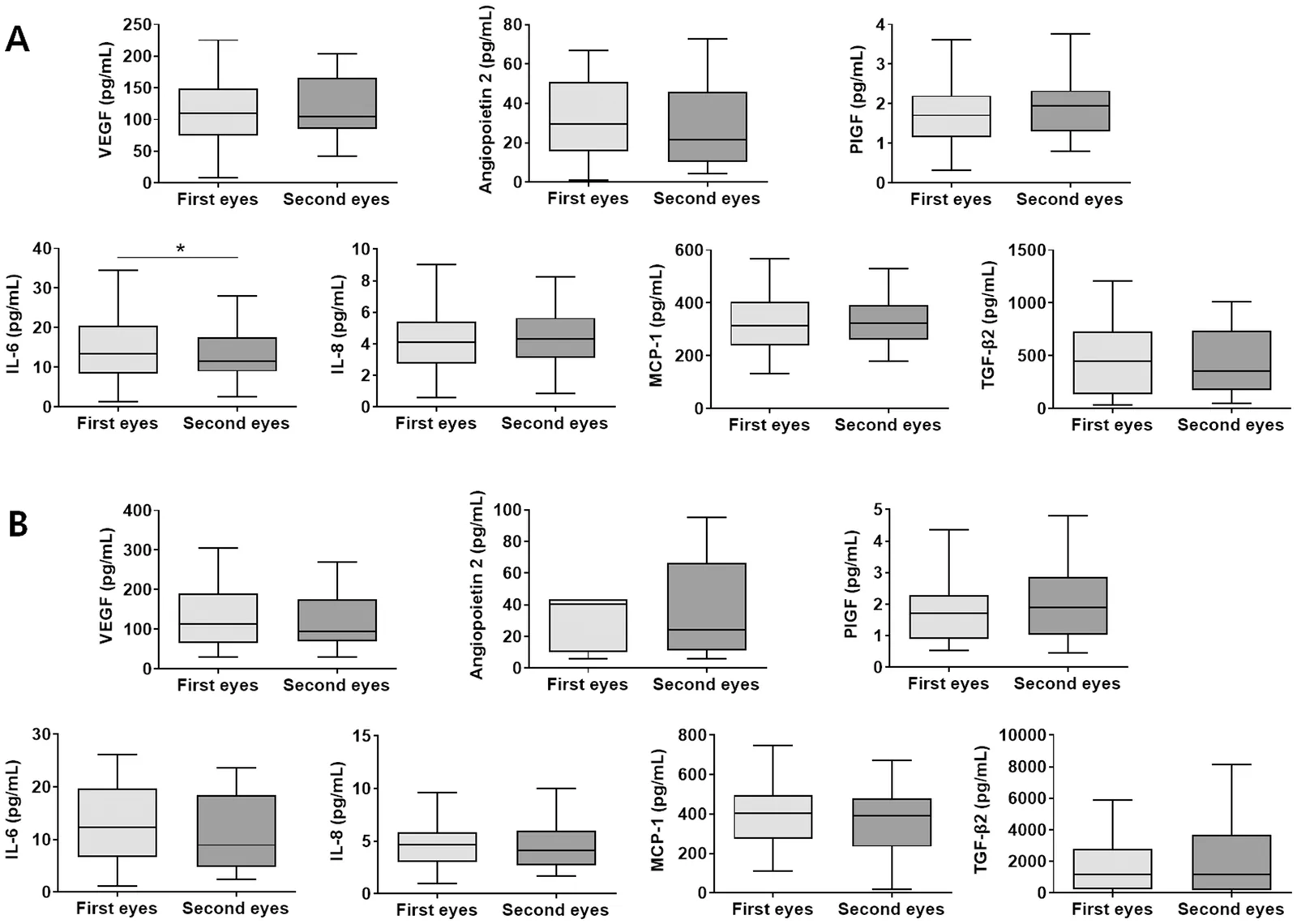
Comparison of cytokine concentrations in the aqueous humor samples of the first and second eyes in the patients (A) without diabetes and (B) with diabetes. Box plots show the median, interquartile range, and Tukey whiskers of cytokine concentrations in the first eyes (gray) and second eyes (dark gray). IL, interleukin; MCP-1, monocyte chemoattractant protein 1; TGF-β2, transforming growth factor beta 2; VEGF, Vascular endothelial growth factor. *Nominal P < 0.05.

In an exploratory analysis among patients without DM, the surgical interval was not significantly associated with the first- versus second-eye difference in IL-6 concentration (Spearman’s ρ = 0.137, P = 0.433). In contrast, there were no significant differences in cytokine concentrations between the first and second eyes of patients with DM (Fig 1B).

## Discussion

In this study, mean IL-6 concentrations were nominally lower in the AH of the second eye than in the first eye among patients without DM, whereas no statistically significant first- versus second-eye difference was observed in patients with DM. However, the formal eye-order × DM interaction was not statistically significant, and the IL-6 finding in patients without DM did not remain significant after FDR adjustment. Therefore, these findings should be considered exploratory, while the observed pattern may warrant further investigation in larger studies. In addition, patients with DM exhibited higher AH concentrations of MCP-1 and TGF-β2 than those without DM. To the best of our knowledge, this is the first reported study to specifically investigate the effect of DM on AH cytokine profiles during sequential bilateral cataract surgery. In order to eliminate the potential confounding effects of DR, we included only patients with DM who had no evidence of DR.

The concept that the two eyes are immunologically interconnected is exemplified by sympathetic ophthalmia, a rare, diffuse granulomatous panuveitis that typically develops in both eyes following unilateral ocular surgery or penetrating trauma [15]. In parallel, patients undergoing second-eye cataract surgery under topical anesthesia frequently report greater intraoperative pain than those undergoing first-eye surgery [10]. Several studies have suggested that this phenomenon is associated with alterations in AH cytokine profiles in the fellow eye, including elevated concentrations of pain-related cytokines such as MCP-1, TNF-α, and IL-1β [11–13]. The authors of these studies proposed that the contralateral elevation of inflammatory cytokines following first-eye surgery may represent a sympathetic ophthalmia-like immune phenomenon, although this hypothesis has not been mechanistically established. Conversely, a recent study on sequential implantable collamer lens (ICL) surgery reported reduced AH concentrations of IFN-γ, IL-4, IL-13, and IL-17A in the second eye compared with the first eye [16]. The authors proposed that the second eye may develop a protective immunoregulatory response following first-eye surgery, although the underlying mechanisms remain uncertain.

IL-6 is a pleiotropic cytokine involved in immunomodulation, inflammation, increased vascular permeability, hematopoiesis, and cell proliferation. In the eye, however, IL-6 is predominantly regarded as a pro-inflammatory cytokine and has been implicated in the pathogenesis of various ocular inflammatory and retinal diseases [17]. The present study found that mean IL-6 concentrations were nominally lower in the AH of the second eye than in the first eye among patients without DM. Although this finding did not remain statistically significant after FDR adjustment and should therefore be regarded as exploratory, the direction of the observed difference may warrant further investigation. Importantly, because baseline AH samples from the second eye before first-eye surgery were unavailable, it cannot be determined whether the observed difference reflects pre-existing inter-eye variability or a biological response associated with first-eye surgery. Given the observational design of this study, the underlying biological mechanisms remain uncertain. Consequently, the observed difference in mean IL-6 concentrations should be interpreted as a correlative finding rather than definitive evidence of a causal, protective anti-inflammatory mechanism. Alternative explanations, including biological variability, regression toward the mean, and systemic influences, cannot be excluded. Nevertheless, the direction of this finding aligns with a recent study of sequential ICL surgery, which similarly reported reduced concentrations of several cytokines involved in inflammatory and immune responses in the second eye and proposed a protective immunoregulatory response following first-eye surgery [16]. Therefore, further studies are needed to determine whether these observations share common biological mechanisms.

In patients with DM, no statistically significant first- versus second-eye difference in IL-6 concentration was observed. In addition, higher MCP-1 and TGF-β2 concentrations were observed in the AH of patients with DM. Although the statistical evidence for these differences was attenuated after FDR adjustment, the observed direction of the differences was generally consistent with previous reports of altered AH cytokine profiles in patients with DM. Recent studies have shown that IL-1β, IL-6, IL-8, MCP-1, VEGF, macrophage inflammatory protein (MIP)-1α, MIP-1β, TGF-β1, and TGF-β2 concentrations are elevated in the AH of patients with DM [7,8,18–20], whereas IL-2, IL-10, IL-12, TNF-α, and interferon-α concentrations are decreased [18].

Collectively, our findings and those of previous studies suggest that diabetic eyes have a chronically altered inflammatory milieu. The higher MCP-1 and TGF-β2 concentrations observed in patients with DM may reflect a pre-existing diabetic inflammatory state. Although the eye-order × DM interaction for IL-6 was not statistically significant, the observed differences in cytokine profiles according to DM status may warrant further investigation in larger studies. The present study design precludes us from distinguishing the relative contributions of these individual factors.

The clinical significance of the elevated individual cytokines should also be interpreted with caution. For instance, MCP-1 has been consistently reported to be elevated in various ocular inflammatory and retinal diseases [21]. Therefore, the higher MCP-1 concentration observed in patients with DM may reflect generalized ocular inflammation rather than a diabetes-specific inflammatory response. Likewise, TGF-β2 is the predominant transforming growth factor isoform in AH and serves as a key mediator of ocular immune privilege through its immunosuppressive effects on intraocular immune responses [22,23]. Beyond its immunoregulatory role, TGF-β2 also regulates extracellular matrix remodeling, fibrosis, and wound healing in ocular tissues [15]. Therefore, elevated TGF-β2 concentrations should not be directly equated with persistent inflammation, as they may reflect multiple biological processes that could not be distinguished in the present study. Because postoperative clinical outcomes and intraoperative pain were not evaluated, the clinical significance of the observed cytokine differences, including their potential associations with postoperative inflammation, cystoid macular edema, and pain perception during second-eye cataract surgery, remains unknown and warrants further investigation.

Several limitations of this study should be acknowledged. First, AH from the second eye was not collected before the first-eye surgery, limiting our ability to directly compare cytokine levels in the second eye before and after surgery. Thus, it cannot be determined whether the observed differences are associated with first-eye surgery or reflect pre-existing inter-eye variability. Accordingly, the observed differences between first- and second-operated eyes cannot be interpreted as true postoperative changes in cytokine concentrations in the second eye. Second, multiple cytokines were evaluated, raising the possibility of type I error due to multiple comparisons. We therefore additionally applied the Benjamini–Hochberg FDR procedure. Several nominally significant findings did not remain statistically significant after FDR adjustment. Accordingly, these findings should be considered exploratory and interpreted cautiously, and further studies are warranted to confirm the observed cytokine differences. Third, only seven preselected cytokines were evaluated. A previous large-scale analysis of aqueous humor immune mediator profiles using machine-learning approaches has demonstrated that multiple immune mediators contribute to the characterization of intraocular diseases [23]. Because intraocular inflammatory responses involve complex cytokine interaction networks, the limited cytokine panel used in this study may not fully capture the immune alterations associated with sequential bilateral cataract surgery. Fourth, although the clinical characteristics of diabetes, including HbA1c levels, diabetes duration, treatment modality, and renal status, are summarized in S1 Table, their associations with AH cytokine concentrations were not evaluated because of the limited sample size. Therefore, residual confounding related to these diabetes-related factors cannot be excluded. In addition, other systemic factors that may influence AH cytokine profiles, such as systemic inflammatory diseases or the use of anti-inflammatory medications, were not evaluated. In addition, postoperative inflammation, cystoid macular edema, and pain were not systematically assessed; therefore, the clinical significance of the observed cytokine differences remains uncertain. Finally, although we restricted inclusion to patients who underwent second-eye surgery within 2 weeks to reduce variability related to surgical interval, the potential influence of the interval between surgeries on AH cytokine concentrations cannot be completely excluded. Although the exploratory analysis showed no significant association between the surgical interval and the first- versus second-eye difference in IL-6 concentration among patients without DM, the limited sample size and restricted range of surgical intervals preclude definitive conclusions regarding interval-dependent differences in cytokine concentrations.

In conclusion, mean AH IL-6 concentrations were lower in the second eye than in the first eye among patients without DM, whereas no statistically significant first- versus second-eye difference was observed in patients with DM. Patients with DM also exhibited higher AH MCP-1 and TGF-β2 concentrations than those without DM. Although some of these findings did not remain statistically significant after FDR adjustment and should therefore be considered exploratory, the eye-order × DM interaction for IL-6 was not statistically significant. The biological mechanisms underlying these observations and their clinical significance remain to be determined through further studies. Because baseline AH cytokine levels in the second eye before first-eye surgery were unavailable, the observed inter-eye differences cannot be definitively attributed to first-eye surgery.

## Supporting information

S1 Table Characteristics of diabetic patients. (DOCX)

S2 Data De-identified individual-level dataset used in the analyses. The dataset includes demographic and clinical characteristics and aqueous humor cytokine measurements from the first and second operated eyes of 71 patients. Missing measurements are retained as missing values. Variable definitions are provided in a separate worksheet.
(XLSX)
