# Supplementary material for: Cytokine profiles of aqueous humor from sequential bilateral cataract surgery: Impact of diabetes on inflammatory responses
Source: PLoS One. 2026 Nov 2;21(11):e0361977. doi: 10.1371/journal.pone.0361977 (PMC13726958; doi:10.1371/journal.pone.0361977)
Supplement: Supplementary file 1 [file pone.0361977.s001.docx]

**S1 Table. Characteristics of diabetic patients**

| **Variable** | **DM (n=32)** |
| --- | --- |
| **Duration of DM (years)** | 10.31 ± 9.11 |
| **HbA1c (%)** | 7.18 ± 1.86 |
| **Fasting glucose (mg/dL)** | 127.78 ± 25.53 |
| **Oral hypoglycemic agents (n, %)** | 30 (93.75 %) |
| **Insulin treatment (n, %)** | 4 (12.50%) |
| **Hypertension (n, %)** | 19 (59.38%) |
| **CKD (n, %)** | 3 (9.38%) |
| **Diabetic nephropathy (n, %)** | 1 (3.13%) |

Data are presented as mean ± standard deviation or n (%).

CKD, chronic kidney disease; DM, diabetes mellitus; HbA1c, glycated hemoglobin.
